# Kinetic Characterization of Tar Reforming on Commercial Ni-Catalyst Pellets Used for In Situ Syngas Cleaning in Biomass Gasification: Experiments and Simulations under Process Conditions

**DOI:** 10.1021/acs.iecr.0c05131

**Published:** 2020-12-16

**Authors:** Andrea Di Giuliano, Pier Ugo Foscolo, Andrea Di Carlo, Andrew Steele, Katia Gallucci

**Affiliations:** † University of L’Aquila, Department of Industrial and Computer Engineering and Economics, Piazzale E. Pontieri 1 - loc. Monteluco di Roio, 67100 L’Aquila, Italy; ‡ Johnson Matthey Technology Centre (JMTC), Blount’s Court, Sonning Common, Reading RG4 9NH, United Kingdom

## Abstract

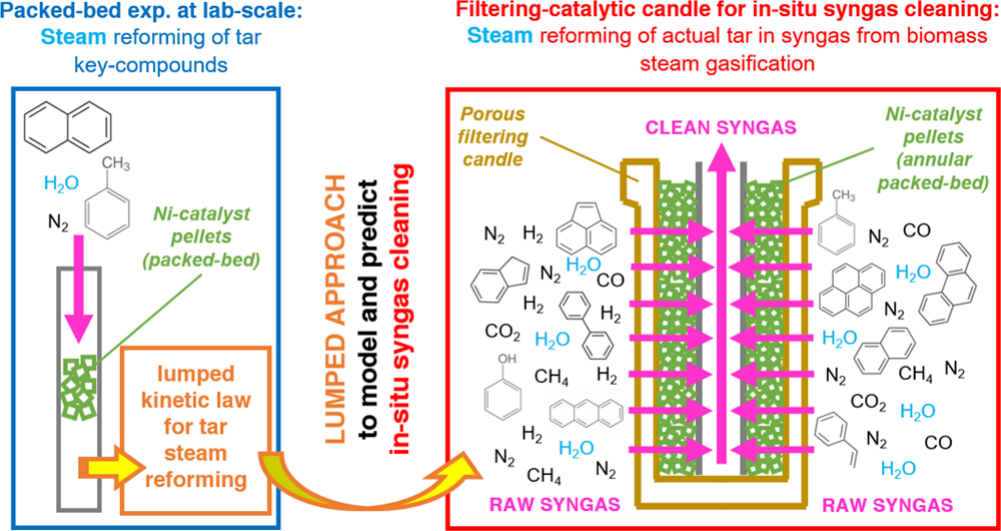

Filtering-catalytic candles, filled with an annular packed-bed of commercial Ni-catalyst pellets (∼600 g), were successfully tested for in situ syngas cleaning in a fluidized-bed biomass steam gasifier [Fuel Process. Technol.2019, 191, 44−53, DOI: 10.1016/j.fuproc.2019.03.018]. Those tests enabled the macroscopic evaluation of gasification and gas cleaning as a whole, requiring a more specific assessment of the catalyst performance inside the filter candle. To this end, steam reforming tests of tar key compounds (naphthalene and toluene; thiophene in traces to observe sulfur deactivation) were performed with a laboratory-scale packed-bed reactor containing the same catalyst pellets (<7 g). A lumped kinetics was derived, referred to a pseudocomponent representing tars. This was then validated by simulation of the annular catalytic packed bed inside the filter candle, obtaining numerical results in fair agreement with gasifier outputs. As a result, the lab-scale investigation with a small amount of catalyst provides reliable predictions of tar catalytic reforming in industrial-scale filtering-catalytic candles.

## 1 Introduction

Biomass attracted the attention of researchers and industry for applications in energy and biofuels production (e.g., methanol, ethanol, mixed alcohols, dimethyl ether, synthetic natural gas, and hydrogen).^1−4^ This interest was also driven by several governmental programs, promoting the use of renewable sources and biofuels:^1^ The European Union (EU) set the goal of a 10% share of biofuels in the transport industry by 2020;^5^ in the USA, the production of biofuels is expected to reach 36 billion gallons by 2022.^6^ This kind of policy, which has continued in the EU by the passing of European green deal,^7^ might represent in the near future a viable means for economic growth, as well as a necessary approach to face the issues related to climate change.^1,8^

Steam gasification of biomass is a relevant route to produce syngas, and then biofuels, with a reduced environmental footprint;^1^ however, the cleaning of raw syngas, mainly consisting of removal of particulate and tar, is a key step of the biomass-to-fuel chain, which has not been fully developed yet.^9,10^ This work deals with the issue of tar removal.

A fluidized-bed gasifier, using biomass as a fuel, produces tars in the order of magnitude of a few g Nm^–3^,^9^ which leave the reactor in the form of vapors or aerosol, along with main gaseous products (H_2_, CO, CO_2_, CH_4_, and H_2_O).^11^ Tar compounds condense by quenching at cold points downstream of gasification and can evolve in more complex molecules by polymerization, therefore increasing the difficulty of removal treatments.^11^ This causes several drawbacks in downstream units: corrosion and fouling of heat exchangers and turbines, deactivation of catalysts in secondary reactors, and clogging of porous components in fuel cells.^12^ Moreover, the formation of tarry molecules constitutes an inefficiency as regards the gasification of the biomass carbonaceous matrix, therefore depleting the syngas yield per unit mass of biomass. In this regard, catalytic steam reforming seems to be the best way to eliminate tar compounds, converting them into additional syngas and thus recovering their energy content, while reducing the amount of pollutants in gasification products.^13,14^

Filtering-catalytic candles were proposed as an innovative, energy-efficient and cost-effective solution to face the issue of tar removal.^11^ These candles may be directly placed inside the freeboard of a fluidized-bed steam gasifier, acting simultaneously as an efficient particulate filter and a catalyst for tar decomposition by steam reforming.^11^ The incorporation of this kind of device inside the gasifier brings in two main advantages:^11,15,16^ the thermal integration of gasification and cleaning operations and the simplification of syngas cleaning and conditioning. The upgrading of raw syngas is performed in situ, with remarkable process intensification of downstream gas treatments in relation to current practice of low-temperature physical/chemical treatments for tar removal.^17,18^

Candles developed so far were made of an anisotropic porous support impregnated with nickel (Ni) and/or integrated with a Ni-based ceramic foam: The particulate filtration was ensured by an external layer with pores of sufficiently low size; the Ni-catalytic phase was able to reduce the tar content from a few g Nm^–3^ down to less than 0.2 g Nm^–3^.^15,19−22^ Experimental studies performed on steam gasification of lignocellulosic biomass revealed that tar is made of several aromatic hydrocarbons. However, in most cases, toluene and naphthalene largely prevail, and after a hot gas catalytic conditioning treatment, these are almost entirely responsible for the remaining tar content in the syngas.^14,16,20,23^ As a matter of fact, heavier aromatics (≥3 rings) were easily reformed on a Ni-based catalytic phase, while toluene and naphthalene were the most recalcitrant toward a similar reforming treatment, among lighter tar components (1 or 2 rings).^16^

Recently, in order to avoid the constraints related to availability and practicability of Ni-impregnated candles, Savuto et al.^24^ proposed a new, simpler concept to realize filtering-catalytic devices: a plain ceramic candle made of porous alumina, filled with pellets of a commercial Ni catalyst developed for hydrocarbons reforming.^24^ They successfully tested this new kind of candle, with real syngas in a pilot-scale fluidized-bed reactor for biomass steam gasification.^24^ In those tests,^24^ the experimental measurements allowed only mass balances which considered the fluidized bed and the candle as a whole, so a specific conclusion could not be made on the performance of Ni catalyst pellets inside the candle. In addition, as a general observation, the simultaneous gasification and syngas cleaning involve a high number of process variables and a complex sequence of phenomena, which both hinder a deeper insight into the intrinsic behavior of catalyst pellets placed inside the candle. In this last regard, the scale of the gasification experiments is an additional considerable factor: If the gasifier is large enough to host a commercial candle, then the related experimental results may be affected by a certain range of variability in the operating boundary conditions. This variability is surely wider than that of dedicated tests at laboratory-scale, focused on the catalytic activity.

This work aims to fill this gap by three complementary approaches: (i) investigating the activity of the same Ni-based catalyst pellets utilized by Savuto et al.,^24^ by means of a laboratory-scale packed-bed reactor rig for the steam reforming of synthetic tar mixtures; (ii) inferring a lumped kinetic law for tar steam reforming, assuming a generic tar mixture to be represented by a carbonaceous pseudocomponent (*C*_tar_), the carbon atoms of which are involved in the steam reforming process; and (iii) validating the kinetic model so developed, by simulations of the behavior of a full scale filtering-catalytic candle segment, placed in the gasifier freeboard.

As far as point (i) is concerned, the reduction of the experimental scale brings in a better control and knowledge of conditions at which the Ni-catalyst pellets operate. The mass of the catalytic bed inside the filtering-catalytic candle segment is about 600 g, made of pellets distributed over a height of about 40 cm.^24^ As a consequence, the catalyst contained in the filter candle, placed in the gasifier freeboard, operates at a not-well-defined temperature distribution, surely in the range between the gasification temperature (i.e., the fluidized bed temperature) and that of syngas exiting at the top of the reactor (evaluated to be about 60 K less).^24^ In contrast, the laboratory-scale packed-bed reactor requires a much smaller amount of catalyst (<7 g), so the pellets are confined in a small reactor volume at a well-controlled temperature. Furthermore, the use of the laboratory-scale rig ensures the additional advantage of complete knowledge and control of inlet conditions. Several parameters were varied in experiments with the packed-bed rig at laboratory scale (inlet tar concentration between 10 and 30 g Nm^–3^ dry, temperature between 700 and 800 °C, sulfur contamination equivalent to 40 or 100 ppm_v_ H_2_S), in order to obtain a kinetic law over a sufficiently wide-range of conditions to describe the in situ syngas cleaning during real biomass steam gasification.

As to point (ii), it is worth stressing that all experiments at laboratory-scale in this work purposely involved the occurrence of sulfur deactivation of the Ni-catalytic pellets, while a series of similar tests, performed in the absence of sulfur species and with the same catalyst, were already presented in the EUBCE (European biomass conference and exhibition) proceedings by Di Giuliano et al.^25^ The lumping approach described in this work was tuned there to obtain kinetic parameters able to describe *C*_tar_ steam reforming in the absence of sulfur species. This work was addressed to investigate the behavior of commercial Ni-catalytic pellets at conditions closer to those of interest for biomass gasification, where sulfur is brought about by biomass itself and is found either in the ashes and in the product gas as H_2_S and COS (carbonyl sulfide), in small concentrations (from 10 to 100 ppm, usually), although these concentrations were sufficient to affect the activity of Ni catalysts.^26^ A kinetic law was derived by fitting the experimental results, which were extended to take into account the influence of sulfur species on the performance of the catalytic treatment for tar abatement.

To the scope of a conclusive validation, i.e., point (iii), the lumped kinetic law for *C*_tar_ steam reforming was implemented in the balance equations of an annular packed bed, which simulated the catalytic inner packing of the filtering-catalytic candles tested by Savuto et al.;^24^ kinetic laws taken from the literature were used to describe additional reactions occurring in those candles. Numerical simulations provided outcomes in fair agreement with experimental results of syngas cleaning and conditioning in the freeboard of the gasifier, performed elsewhere,^24^ especially as far as tar reforming is concerned.

## 2 Materials and Methods

### 2.1 Commercial Ni-Catalyst Pellets

Johnson Matthey kindly supplied the commercial catalyst pellets utilized in this work, together with density specification. These pellets have a cylindrical shape: 3 mm wide and 3 mm high. This small size allowed them to be used in both the packed-bed rig described in section 2.2 and the full-scale filtering-catalytic candles studied by Savuto et al.^24^ for in situ syngas cleaning.

### 2.2 Tar Steam Reforming Tests at Laboratory Scale

**Figure 1:**
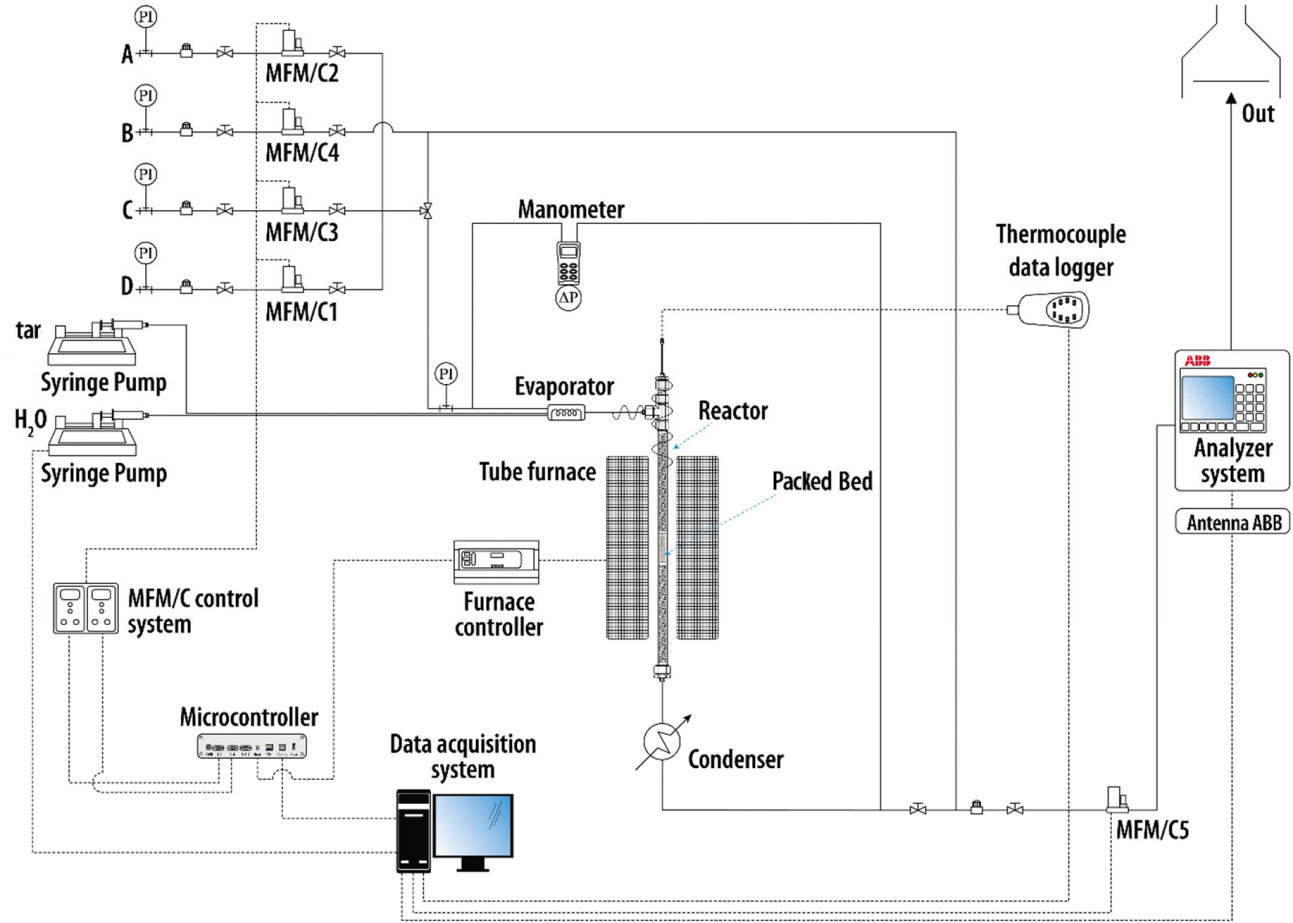
Schematic view of the packed-bed rig for tar steam reforming tests.

A packed-bed rig at laboratory scale (Figure 1) was used to study tar steam reforming on commercial Ni-catalyst pellets.

The experimental rig consisted of a vertical stainless-steel pipe (internal diameter of 1.6 cm, 0.5 m long), heated by a cylindrical electrical furnace. The catalytic active packed bed (3.9 or 6.5 g) was placed at middle height, in the central part of the furnace, ensuring the best temperature control.

The thermocouple involved in the control loop had its tip located inside the catalyst bed. Temperatures of 800, 750, and 700 °C were investigated, as they constitute a range of interest for the in situ syngas cleaning by filtering-catalytic candles.

Two stainless-steel pressure syringes, driven by electric engines (KDS LEGATO 110), pumped water and a liquid synthetic solution of tar key compounds into a vaporization chamber at 220 °C. This pumping system controlled their volumetric flow; in order to compile mass balances for each test, the density of the pumped synthetic tar solution was determined by a pycnometer.

This solution was made up of toluene (0.77 molar fraction), naphthalene (0.21 molar fraction), and a minor fraction of thiophene (0.02 molar fraction). The toluene/naphthalene molar ratio was 3.7, close to naphthalene solubility in toluene at ambient temperature;^27^ this ensured the synthetic tar feed to be liquid, without any solid precipitate which could clog the syringe pump. Thiophene was added to investigate the reversible deactivation of Ni catalyst due to the sulfur species present at low concentrations. In addition, the toluene/naphthalene ratio utilized in these experiments was on the same order of that found in the product gas of steam biomass gasification tests, before any catalytic treatment.^20,23,24,28^

N_2_ was fed to the vaporization chamber as a carrier gas (600 or 780 NL min^–1^), in order to convey vaporized fluids to the reactor. This inert gas stream simulated the flow rate of the actual syngas, in order to allow the specific quantification of tar conversion due to catalytic steam reforming, by means of a dedicated carbon balance.

Proper inlet flow rates were set for liquids and gases to make the content of steam, heavy hydrocarbons, and sulfur species compatible with those of the raw syngas produced during the biomass gasification tests of Savuto et al.^24^ and to obtain realistic contact times between inlet gas stream and catalytic bed. The inlet steam to carbon molar ratio ranged between 6.6 and 19.9, with H_2_O always being in a large stoichiometric excess with respect to tar key compounds in the synthetic mixture, as far as steam reforming and water gas shift (WGS) are concerned. The inlet molar N_2_ to steam ratio was equal to 2.3 or 3. The concentration of tar key compounds was varied between 10 and 30 g Nm^–3^_dry_.

This setting of flow rates allowed thiophene to be fed in such a quantity to develop 40 or 100 ppm_v_ equivalent H_2_S in the inlet stream (1:1 atomic ratio of S between thiophene and H_2_S, assuming the complete conversion to H_2_S because of the high excess of steam and the reductive environment developed inside the packed-bed rig). Ma et al.^29^ found that the sulfur deactivation of Ni is surely reversible up to H_2_S concentration of 200 ppm at process conditions similar to those operated in this work, as only physical adsorption of H_2_S occurs on Ni catalytic sites. Depner and Jess^30^ investigated the tar steam reforming on a commercial Ni catalyst and determined the upper limit of reversible H_2_S deactivation at 0.1 vol % H_2_S; for higher concentrations, Ni-sulfide formation was reported. Along the lines of these findings, in this work it is assumed to deal with a reversible deactivation of the commercial Ni catalyst.

Downstream, a glass double-pipe condenser separated unreacted water and condensable hydrocarbons from the product stream, with ethylene glycol at 0 °C as the cooling fluid. The dried outlet stream passed through a Bronkhorst mass flow meter, which measured the overall molar flow rate (*F*_tot,out_). Then, this outlet stream was analyzed by an ABB online system, which measured volumetric percentages (*y*_*i*,out_) of CO, CO_2_, CH_4_, and H_2_. This system was equipped with an Advance Optima Uras 14 module for CO, CO_2_, and CH_4_ (nondispersive infrared detector) and an Advance Optima Caldos 17 module for H_2_ (thermal conductibility detector). Values of *F*_tot,out_ and *y*_*i*,out_ were recorded for a sampling period of 5 s and allowed the calculation of outlet molar flow rates (*F*_*i*,out_, eq 1) and percentages on dry, dilution-free basis (*Y*_*i*,out_, eq 2) of CO, CO_2_, CH_4_, and H_2_.
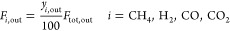
1
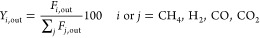
2Before each experiment, the catalytic pellets were prereduced in order to obtain Ni^0^, the actual catalytic active phase for reforming:^31−33^ A heating ramp at 10 °C min^–1^ was operated from room temperature up to 900 °C, followed by a 30 min dwell at 900 °C, while 150 NmL min^–1^ of a reducing stream (10 vol % H_2_ in N_2_) flowed through the packed bed. Each reforming step lasted long enough to observe the stabilization of product formation, in such a way as to record an adequate amount of data under steady-state conditions and to be sure that the reversible sulfur deactivation occurred completely. Reforming durations were also comparable to those of gasification tests with filtering-catalytic candles carried out by Savuto et al.,^24^ the results of which were used in this work as a reference for the final validation procedure.

### 2.3 Lumped Kinetic Law for Tar Steam Reforming

#### 2.3.1 Tar Mixture As a Monocarbonic Pseudocomponent

What is usually referred as “tar” is actually a complex mixture of diversified, condensable hydrocarbons with molecular weights larger than that of benzene.^34,35^ These molecules range from 1 to 7 aromatic rings, divided in five classes (Table 1).^36^

**Table 1:** Classification of Tar Components^a^

group	name	composition
class 1	GC-undetectable	determined by subtracting the GC-detectable tar fraction from the total gravimetric tar.
class 2	heterocyclic aromatics	e.g., pyridine, phenol, cresol, and quinoline
class 3	aromatics (1 ring)	e.g., xylene, styrene, and toluene
class 4	light PAH compounds (2–3 rings)	e.g., naphthalene, biphenyl, acenaphtylene, fluorene, phenanthrene, and anthracene
class 5	heavy PAH (4–7 rings)	e.g., fluoranthene, pyrene, chrysene, benzo-fluoranthene, benzopyrene, and perylene

a GC = gas-chromatograph. PAH = polyaromatic hydrocarbons, adapted from ref (36).

The yield of formation of tar components depends on the kind of thermochemical conversion (e.g., pyrolysis, steam gasification, and partial oxidation), the kind of fuel to be converted (e.g., biomasses and coal), and the process conditions (e.g., temperature).^37^ In addition, for a given process, tar can include a large number of chemical species from the five classes in Table 1. As a consequence, the study of tar behavior may turn out to be tricky if each hydrocarbon must be individually traced. To overcome these constraints, synthetic mixtures of tarry molecules are usually investigated in laboratory-scale studies, made up of a few species which are supposed to mimic the behavior of the real tar developed in an actual thermochemical process. However, the transfer of information from these kind of experiments to actual tar mixtures may yield questionable results when the composition of actual tar from a thermochemical process is much more complex than that of synthetic tar mixtures. Usually, in synthetic tar mixtures, naphthalene and toluene are chosen as tar key compounds,^29,30,38,39^ since they are the most abundant and also those responsible for the remaining tar content in the product syngas, after a catalytic hot gas cleaning treatment (as mentioned in the Introduction).^16,20,23^

This section proposes a general procedure to simplify the transfer of information from experimental campaigns with synthetic tar mixtures toward actual processes, as far as the main interest concerns the overall behavior of tar as a whole.

Let us consider a generic tar mixture made up of *N* hydrocarbons with the generic chemical formula C_*n*_H_*m*_ (other kinds of atoms in actual hydrocarbons are considered negligible, as far as the purposes of the lumping procedure are concerned). Molar fractions of these hydrocarbons (*x*_tar__,*i*_ for the *i*th hydrocarbon) are known. The goal of the procedure is the reduction of this mixture into a monocarbonic pseudocomponent, namely, *C*_tar_, and identified by the chemical formula CH_(*h*/*c*)_. The indexes *h* and *c* are calculated by eqs 3 and 4, respectively, where *m*_*i*_ is the H index in the chemical formula C_*n*_H_*m*_ of the *i*th hydrocarbon and *n*_*i*_ is the analogous index for C.
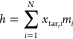
3
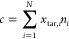
4*C*_tar_ is intended to represent the main average functional group which constitutes tar molecules in a mixture. Therefore, it allows a lumped approach when tar chemical conversion is studied: A unique chemical reaction, with CH_(*h*/*c*)_ as a reactant, substitutes for the set of reactions individually occurring to the *N* hydrocarbons.

#### 2.3.2 Lumped Kinetic Law for Tar Steam Reforming

In this work, the approach described in section 2.3.1 was applied to the steam reforming of tar occurring on Ni-catalyst pellets: Reaction R1 summarizes the *N* steam reforming reactions undergone by the *N* tar components. In any case, steam reforming is accompanied by WGS (reaction R2).
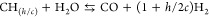
R1
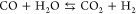
R2Once the formula CH_(*h*/*c*)_ of *C*_tar_ is calculated and its steam reforming is obtained (reaction R1), the definition of a kinetic law for this reaction is the following step.

In agreement with the literature dealing with the steam reforming of tarry molecules,^29,38^ a pseudo-first-order was postulated for reaction R1 (eq 5), with respect to *C*_tar_ molar concentration (*C*_*C*_tar__), while the kinetic dependency on water was not considered because of its large excess in comparison to stoichiometric ratios. To take into account the reversible sulfur deactivation of Ni catalyst, an adsorption term (*K*_S_) was introduced in the kinetic law (eq 6), as done by Ma et al.^29^ The dependences on temperature were expressed by the Arrhenius equation for the specific rate of *C*_tar_ steam reforming (eq 7), by a van ’t Hoff-type relation for sulfur species adsorption (eq 8). The treatment as an adsorption function for the sulfur deactivation term in eq 8 agrees with the mechanism assumed for Ni deactivation at the reforming experimental condition of this work (see section 2.2); Depner and Jess^30^ found that the mathematic structure of eq 8 also fits well the deactivation on Ni catalyst at H_2_S concentration higher than 0.1 vol %, when Ni-sulfides are formed, even though in this case it should be considered only as a fairly good mathematical description of the H_2_S influence on reaction rates, losing the physical meaning of an adsorption term.
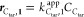
5
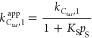
6
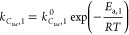
7
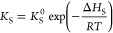
8

#### 2.3.3 Estimation of Lumped Kinetic Parameters

Under the assumptions of sections 2.3.1 and 2.3.2, data from experiments in the packed bed allowed the estimation of the lumped kinetic parameters.

The catalytic active bed was modeled as a plug flow reactor (PFR) at steady state. The related mole balance for *C*_tar_ (i.e., the pseudomolecule CH_(*h*/*c*)_) was formulated by assuming the kinetic law in eq 5 and expressing the molar concentration *C*_*C*_tar__ in terms of *C*_tar_ conversion (χ_*C*_tar__) and experimentally known quantities such as *h*, *c*, the inlet *C*_tar_ flow rate (*F*_*C*_tar_,in_), the inlet molar steam to carbon ratio (α_in_), and the inlet molar N_2_ to steam ratio (β_in_) (see section S1 of Supporting Information for further details). Equation 9 resulted from this operation and was then properly integrated with respect to the variable packed bed mass (*w*) from 0 to the total mass of pellets (*W*), obtaining the algebraic eq 10. Equations 9 and 10 remain valid when the apparent specific reaction rate of *C*_tar_ reforming (*k*_*C*_tar_,1_^app^) depends on sulfur deactivation (eq 8), since only experimental data in the steady state were considered in this work for kinetic determinations (i.e., after H_2_S adsorption is completed and its concentration can be assumed to be constant throughout the bed).^29^
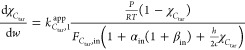
9
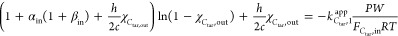
10The conversion of *C*_tar_ at the packed-bed outlet (χ_*C*_tar_,out_) was determined by a carbon balance, as the ratio between total carbon moles which left the reactor as CO_*x*_ (no CH_4_ was detected in the outlet stream, for all tests) and the total carbon moles fed to the reactor (eq 11). For each experiment, that balance was based on data corresponding to a proper time interval (τ in eq 11), during which the process took place in a steady state. This ensured the fulfillment of the hypotheses of eqs 9 and 10, as well as to consider the reversible deactivation due to sulfur as fully developed; in such a way, the partial pressure of sulfur species at the inlet equals that in the packed-bed void fraction (*p*_*S*_).
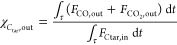
11For each experiment, once χ_*C*_tar__ is obtained from experimental data by eq 11, eq 10 allows the calculation of *k*_*C*_tar_,1_^app^.

As stated in the Introduction, in a preliminary work Di Giuliano et al.^25^ obtained kinetic parameters for the Ni-catalytic pellets provided by Johnson Matthey, characterizing *C*_tar_ steam reforming in the absence of any sulfur deactivation with the same methodology adopted here. Values of the pre-exponential factor *k*_*C*_tar_,1_^0^ and the activation energy *E*_a,1_ were obtained by the regression of related experimental data, based on eq 7 (linearized by logarithmic transformation): *k*_*C*_tar_,1_^0^ and *E*_a,1_ equaled 297 152 m^3^ kg_cat_^–1^ min^–1^ and 105.6 kJ mol^–1^, respectively.^25^ The *E*_a,1_ value was included in the range reported in the literature for the steam reforming of toluene (196 kJ mol^–1^)^38^ and naphthalene (94 kJ mol^–1^)^29^ over Ni-based catalysts, confirming the validity of the procedure.^25^

In this work, new experiments were carried out with sulfur species in the reactor feed; the adsorption term *K*_S_ was calculated by eq 6, thanks to the knowledge of *p*_S_, *k*_*C*_tar_,1_^0^, and *E*_a,1_. The preexponential factor *K*_S_^0^ and the enthalpy of adsorption Δ*H*_S_ were then obtained by regression based on eq 8 (linearized by logarithmic transformation).

## 3 Results and Discussion

### 3.1 Steam Reforming Results

Six tests were carried out, two for each chosen temperature: their inlet and operating conditions are summarized in Table 2.

**Table 2:** Operating and Inlet Conditions of Steam Reforming Tests and Corresponding Experimental Results of χ_*C*_tar__ and Kinetic Constants *k*_*C*_tar_,1_^app^ and *K*_S_

	test 1	test 2	test 3	test 4	test 5	test 6
Process Conditions						
*P* [atm]	1	1	1	1	1	1
*T* [°C]	800	800	750	750	700	700
*W* [g]	3.9	3.9	6.5	6.5	6.5	6.5
Inlet						
*h* [-]	7.92	7.92	7.92	7.92	7.92	7.92
*c* [-]	7.57	7.57	7.57	7.57	7.57	7.57
*F*_N_2_,in_ [NL min^–1^]	600	600	780	780	780	780
tar concentration [g Nm^–3^_dry_]	30.0	10.0	12.8	12.8	12.8	12.8
H_2_S equivalent [ppm_v_]	100	100	40	40	40	40
*F*_*C*_tar_,in_ [mol min^–1^]	1.35 × 10^–2^	4.5 × 10^–3^	7.5 × 10^–3^	7.5 × 10^–3^	7.5 × 10^–3^	7.5 × 10^–3^
α_in_ [mol_H_2_O_ mol_*C*_tar__^–1^]	6.6	19.8	19.9	19.9	19.9	19.9
β_in_ [mol_N_2__ mol_H_2_O_^–1^]	3.0	3.0	2.3	2.3	2.3	2.3
WHSV [mol_in_ h^–1^ kg_cat_^–1^]	570.0	556.1	466.0	466.0	466.0	466.0
WHSV_*C*_tar__ [mol_*C*_tar_,in_ h^–1^ kg_cat_^–1^]	20.8	6.9	6.9	6.9	6.9	6.9
Outlet						
χ_*C*_tar_,out_ [%]	29.2	30.5	38.5	38.2	21.3	21.9
Kinetic Calculations						
*k*_*C*_tar_,1_^app^ [m^3^ kg_cat_^–1^ min^–1^]	0.290	0.297	0.318	0.314	0.149	0.154
*k*_*C*_tar_,1_[m^3^ kg_cat_^–1^ min^–1^]^a^	2.148	2.148	1.205	1.205	0.636	0.636
*K*_S_ [atm^–1^]	64181	62269	69708	70859	82053	78466

a Calculated at *T*, by eq 7 with *k*_*C*_tar_,1_^0^ = 297152 m^3^ kg_cat_^–1^ min^–1^ and *E*_a,1_ = 105.6 kJ mol^–1^.^25^

For all experiments, weight hourly space velocities (WHSV, eq 12) and WHSV referred to *C*_tar_ (WHSV_*C*_tar__, eq 13) were higher than those experienced by Ni-catalyst pellets in the filtering-catalytic candles during hot gas cleaning in the gasifier freeboard of Savuto et al.;^24^ this allowed testing the catalyst in more severe conditions and getting data suitable for the kinetic characterization.
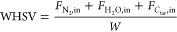
12
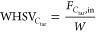
13The two tests at 800 °C (tests 1 and 2) were performed at the same conditions, except for the inlet concentration of synthetic tar, which equaled 30 and 10 g Nm^–3^_dry_, respectively. This variation allowed verifying the assumption of first-order reaction in the lumped kinetic law of *C*_tar_ steam reforming (eq 5). For all other tests, H_2_S equivalent, WHSV, and WHSV_*C*_tar__ were decreased, in such a way to obtain substantial conversions, despite the reaction rate reduction due to the temperature decrease down to 750 and 700 °C (in any case, WHSV and WHSV_*C*_tar__ were still much higher than those in filtering-catalytc candles, as stated above). At each of these temperatures, two tests were repeated with the same conditions, to verify the repeatability of experiments in the packed-bed rig.

**Figure 2:**
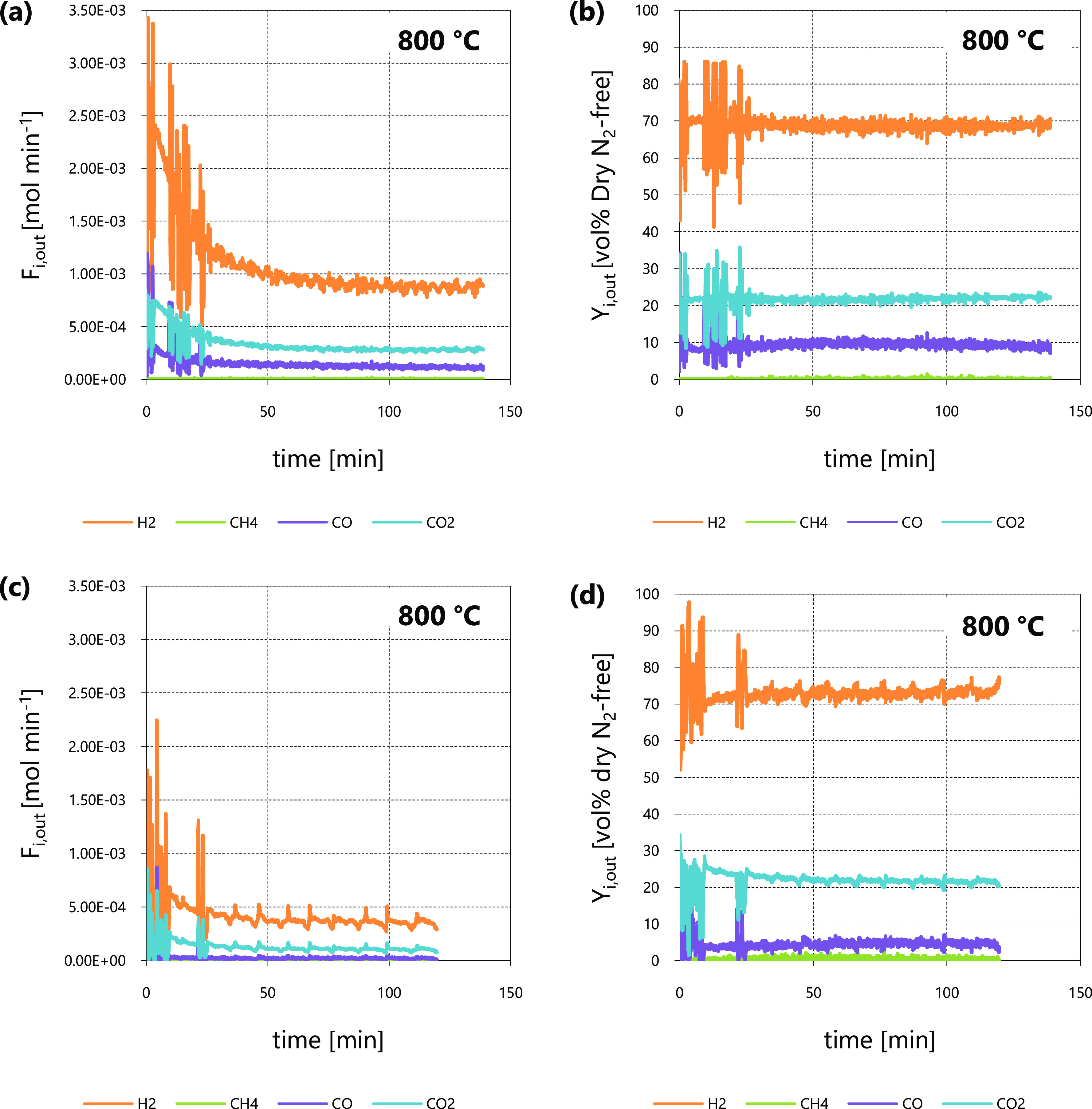
Steam reforming tests at 800 °C: *F*_*i*,out_ (a) and *Y*_*i*,out_ (b) as functions of time from test 1; *F*_*i*,out_ (c) and *Y*_*i*,out_ (d) as functions of time from test 2.

Figure 2 shows the experimental performance of commercial Ni catalyst at 800 °C, during test 1 (Figure 2a,b) and test 2 (Figure 2c,d). Prior to the reforming steps, the packed-beds had just undergone the prereduction procedure (see section 2.2), so the whole trend of catalyst deactivation was observed: a progressive decrease of products molar outlet flow rates (*F*_*i*,out_) occurred, until stabilization after about 100 min (Figure 2a,c). The fluctuations of experimental data in Figure 2, particularly in the first part, should be ascribed to the settling of the syringe pump system. In any case, only sequences of data in the steady state were used for the calculation of *C*_tar_ conversion at the packed-bed outlet (χ_*C*_tar_,out_, eq 11).

Values of χ_*C*_tar_,out_ equaled 29.2 and 30.5% for tests 1 and 2, respectively, notwithstanding the important difference between their inlet tar concentrations (30 and 10 g Nm^–3^ dry gas, Table 2); this behavior confirmed the assumption of first-order kinetics^40^ with reference to *C*_tar_ concentration in eq 5. These values of χ_*C*_tar_,out_ and the tar inlet levels of tests 1 and 2 were in agreement with the observed differences between *F*_*i*,out_ of test 1 (Figure 2a) and test 2 (Figure 2c): With similar conversions, the greater the *C*_tar_ inlet (*F*_*C*_tar_,in_) the higher the flow rates of products from reactions R1 and R2 (*F*_*i*,out_).

As far as outlet molar percentages on dry, diluent-free basis (*Y*_*i*,out_) of test 1 (Figure 2b) and test 2 (Figure 2d) are concerned, their different values can be correlated with process conditions (Table 2): The inlet steam to *C*_tar_ ratio (α_in_) in test 2 was higher, so it pushed the equilibrium of the WGS (reaction R2) toward products more than that in test 1. As a consequence, the outlet H_2_ concentration (*Y*_H_2_,out_) and the ratio between outlet concentrations of CO_2_ and CO (*Y*_CO_2_,out_ and *Y*_CO,out_) from test 2 were greater than those from test 1.

**Figure 3:**
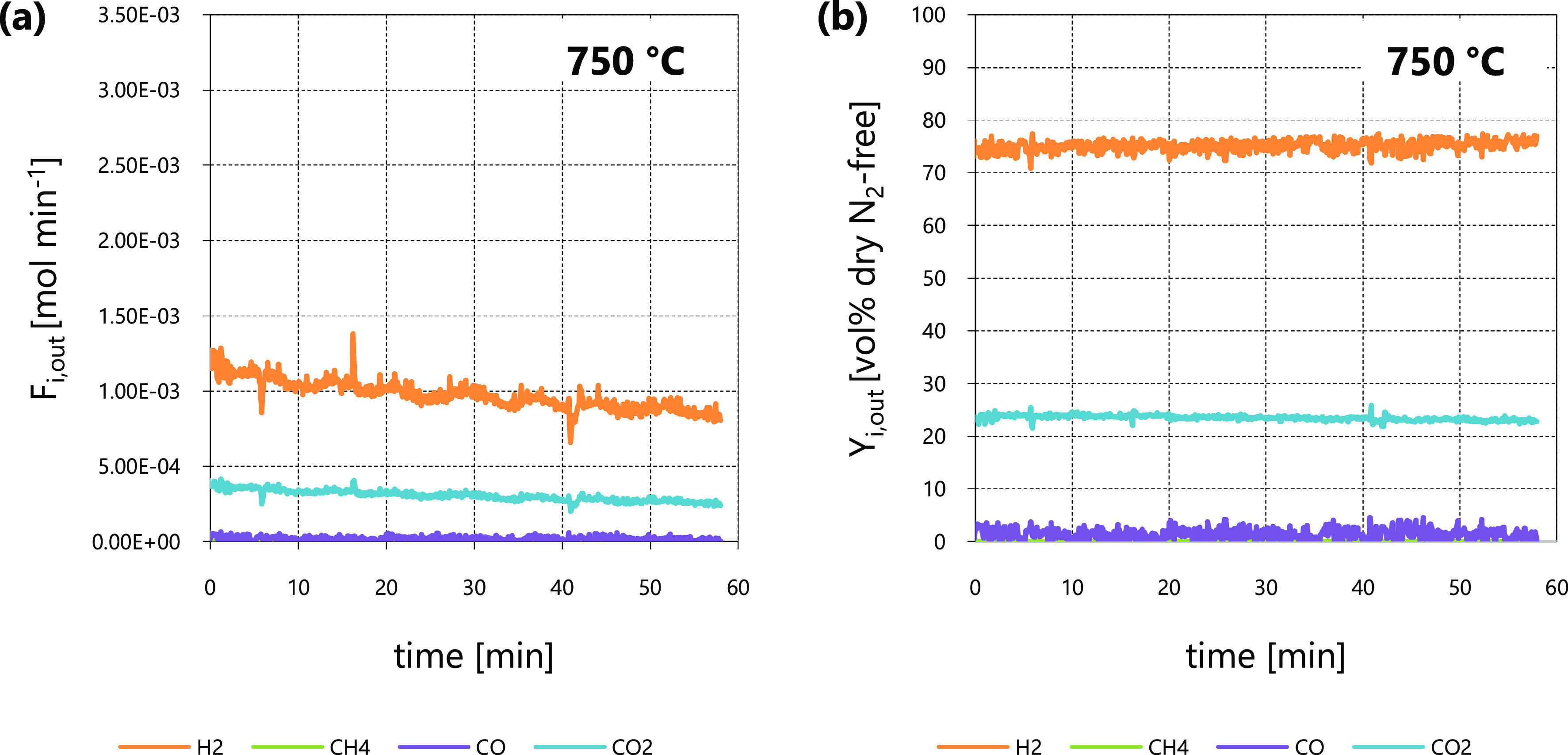
Steam reforming test 4 at 750 °C: *F*_*i*,out_ (a) and *Y*_*i*,out_ (b) as functions of time.

**Figure 4:**
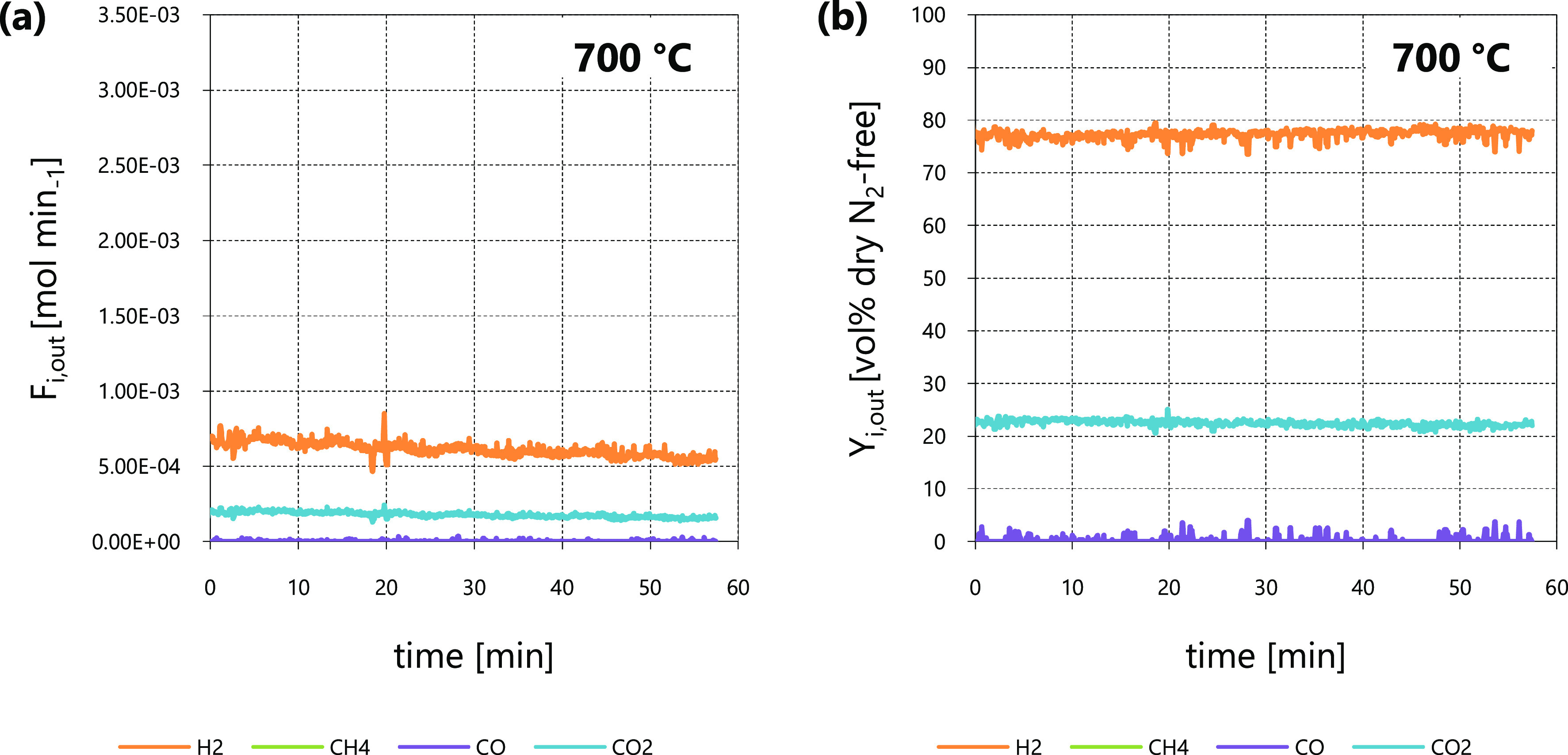
Steam reforming test 6 at 700 °C: *F*_*i*,out_ (a) and *Y*_*i*,out_ (b) as functions of time.

With regard to tar reforming at 750 and 700 °C, the two repeated tests gave very close outcomes in terms of *C*_tar_ conversion at the reactor outlet (χ_*C*_tar_,out_, see Table 2) at each temperature. This proved the repeatability of the reforming experiments discussed in this work, performed with rig and methodology described in section 2.2. For all these experiments, the pre-reduction step and a tar reforming session (800 °C, at least 1 h) preceded the tar reforming at 750 and 700 °C; this preliminary reforming ensured the deactivation of the catalytic bed due to sulfur. As an example of experimental outcomes at 750 and 700 °C, Figures 3 and 4 show outlet molar flow rates (*F*_*i*,out_) and molar percentages on dry dilution-free basis (*Y*_*i*,out_) as functions of time, as obtained from tests 4 and 6, respectively. The *F*_*i*,out_ data from test 4 (Figure 3a) were greater than those from test 6 (Figure 4a). All inlet and operating conditions were the same, with the exception of temperature, so the different magnitudes of outlet molar flow rates *F*_*i*,out_ were ascribed to the influence of temperature on the kinetic law. The higher the temperature, the greater the *F*_*i*,out_ values. As a consequence, the values of *C*_tar_ conversion at packed-bed outlet at 750 °C (χ_*C*_tar_,out_ between 38 and 39%, see Table 2) were higher than those at 700 °C (χ_*C*_tar_,out_ between 21 and 22%, see Table 2). The order of magnitude of χ_*C*_tar_,out_ at 750 and 700 °C was similar to that obtained at 800 °C, so the variation of operating parameters when moving from 800 °C to lower temperatures (Table 2) ensured to keep the outlet molar flow rates *F*_*i*,out_ (and therefore the outlet *C*_tar_ conversion χ_*C*_tar_,out_) within analytically substantial ranges, despite the depletion of reaction rate due to temperature.

At 750 and 700 °C (Figure 3b and 4b, respectively), outlet CO concentration (*Y*_CO,out_) values were lower than those of CO_2_ (*Y*_CO_2_,out_); this was ascribed to the influence on the WGS (reaction R2) equilibrium of the high excess of steam in the reaction environment, which was even higher in comparison to that of tests at 800 °C (compare the respective inlet N_2_ to steam ratios β_in_, Table 2). Despite the very low values of *Y*_CO,out_, differences emerged when comparing results at 750 and 700 °C (Figures 3b and 4b, respectively): The CO fraction reduced its value as temperature was decreased, in agreement with the fact that CO is a reactant involved in the WGS exothermic reaction (reaction R2).

### 3.2 Regression of Kinetic Lumped Parameters

Values of operating and inlet conditions (Table 2) were set in eq 10, together with values of *C*_tar_ conversion at packed-bed outlet (χ_*C*_tar_,out_) obtained experimentally, in order to calculate the corresponding values of the apparent specific reaction rate of *C*_tar_ reforming *k*_*C*_tar_,1_^app^ and then of the adsorption term of sulfur species *K*_S_, as described in section 2.3.3. The results of these calculations are summarized in Table 2.

**Figure 5:**
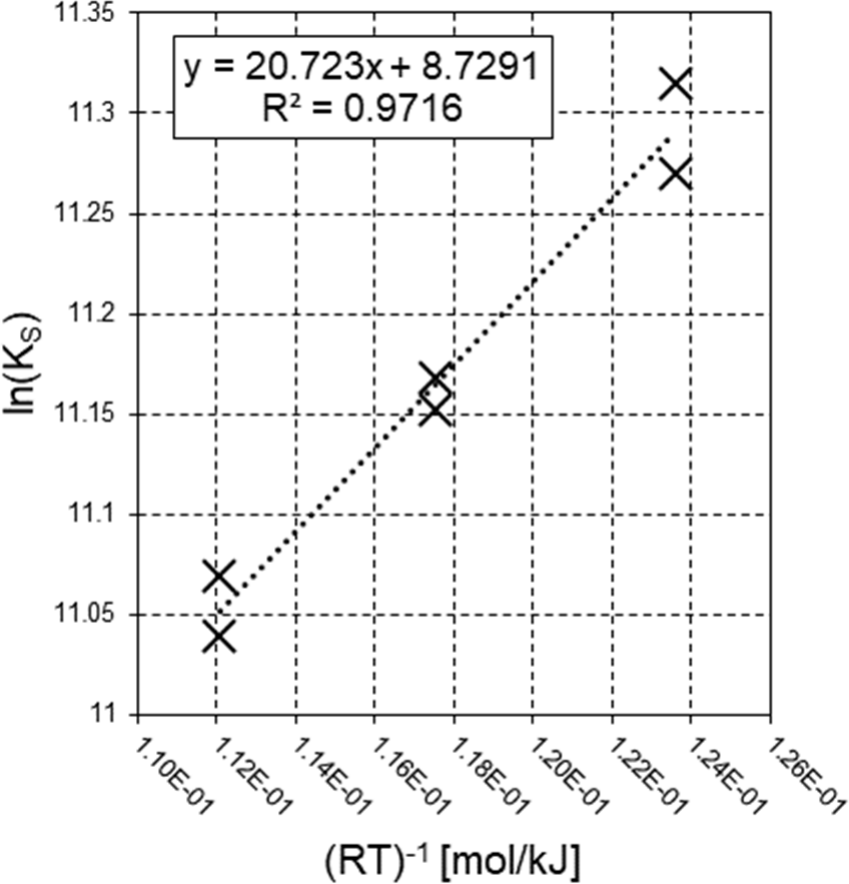
Regression of van ’t Hoff parameters from logarithmically linearized eq 8.

The six experimental values of *K*_S_ were used to perform a linear regression based on eq 8, with (*RT*)^−1^ as the independent variable (Figure 5). In such a way, the slope of the regression line equaled −Δ*H*_*S*_ (enthalpy of adsorption of H_2_S with opposite sign), and its interception point with the vertical axis equaled the natural logarithm of the pre-exponential factor in eq 8, ln(*K*_S_^0^). The quality of the regression was acceptable, as assessed by the value of the coefficient of determination (*R*^2^, Figure 5); the outcomes were *K*_S_^0^ = 6180.2 atm^–1^ and Δ*H*_S_ = −20.7 kJ mol^–1^ (Figure 5).

The negative value of the enthalpy of adsorption of H_2_S on Ni sites (Δ*H*_S_) agrees with the findings from Depner and Jess:^30^ For several hydrocarbons (methane, benzene, and naphthalene) and in a comparable temperature range, they determined that the inhibition by H_2_S on their commercial Ni catalyst (1.5 mm particles) decreased as the temperature was increased. Different Δ*H*_S_ numerical values were found in a given experimental campaign by changing only the hydrocarbon to be reformed,^29,30^ so the value of the adsorption enthalpy Δ*H*_S_, drawn by the regression in Figure 5, should be considered specific for the lumped *C*_tar_ pseudocomponent.

**Figure 6:**
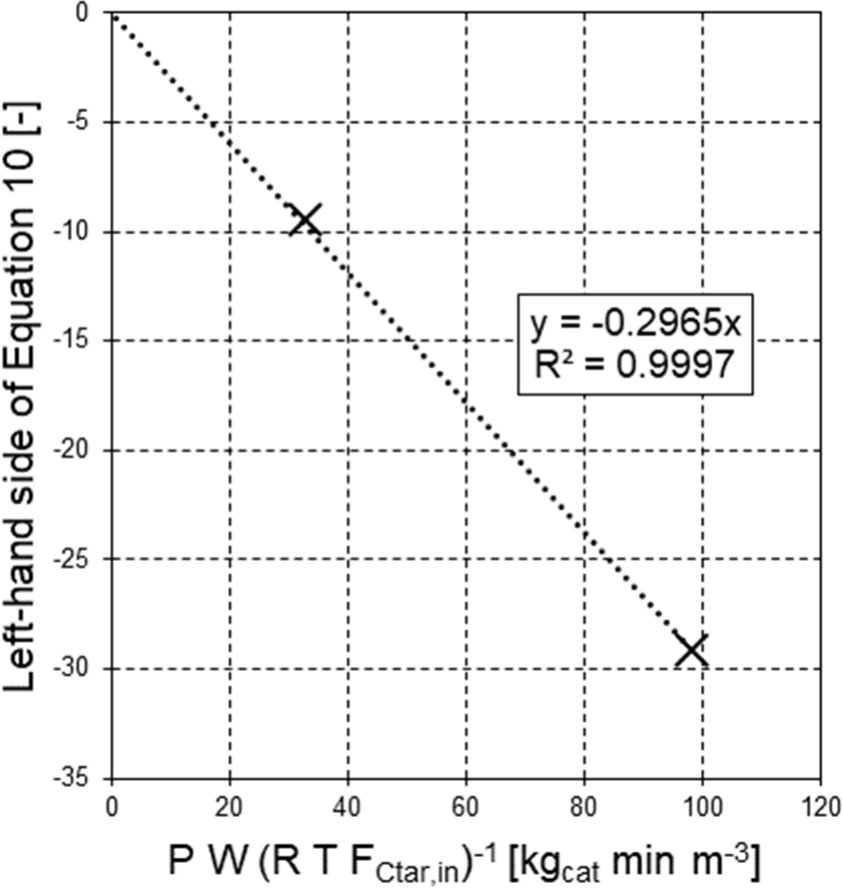
Linear regression of results from tests 1 and 2, imposing the condition that the interception point of the straight line with the vertical axis should be equal to zero, according to eq 10.

As a countercheck, an additional regression was performed (Figure 6), based on the variation of the *C*_tar_ inlet flow rate (*F*_*C*_tar_,in_) in the two experiments at 800 °C. Equation 10 was interpreted as a straight line with *P W*(*F*_*C*_tar_,in_*RT*)^−1^ as the independent variable and its LHS as the dependent variable, so −*k*_*C*_tar_,1_^app^ (i.e., the apparent specific reaction rate of *C*_tar_ steam reforming with opposite sign) became the slope. According to the experimental data in Table 2, values of the dependent variable were calculated for tests 1 and 2, then plotted in Cartesian coordinates as functions of the independent variable, also obtained from Table 2 (Figure 6). It is worth noting that the LHS of eq 10 must be null when *P W*(*F*_*C*_tar_,in_*RT*)^−1^ is zero: As a result, a linear regression is allowed (Figure 6) by imposing that the interception point of the straight line with the vertical axis should be equal to zero. The two experimental points matched well the above condition concerning the interception point with the vertical axis, as they made the coefficient of determination *R*^2^ very close to 1 (Figure 6). In addition, the absolute value of the slope obtained by this operation (0.2965 m^3^ kg_cat_^–1^ min^–1^, Figure 6) was very close to the value of the apparent specific reaction rate of *C*_tar_ steam reforming (*k*_*C*_tar_,1_^app^) derived from tests 1 and 2 in Table 2.

Table 3 summarizes the values of all kinetic parameters obtained experimentally that describe steam reforming of *C*_tar_ on the commercial Ni-catalyst pellets investigated in this work.

**Table 3:** Values of Kinetic Parameters for *C*_tar_ Steam Reforming

*k*_*C*_tar_,1_^0^ [m^3^ kg_cat_^–1^ min^–1^]	297 152
*E*_a,1_ [kJ mol^–1^]	105.6
*K*_S_^0^ [atm^–1^]	6180.2
Δ*H*_S_ [kJ mol^–1^]	–20.7

### 3.3 Validation of Kinetic Parameters with Gas Cleaning in Real Gasification Tests

#### 3.3.1 Modeling the Catalytic Annular Packed-Bed of the Candle

As recalled above, Savuto et al.^24^ successfully tested a device to clean in situ the raw syngas produced by biomass gasification in a fluidized bed. That device consisted of a segment of a commercial inert porous ceramic candle made of Al_2_O_3_, acting as a particulate filter (supplied by PALL Filtersystems GmbH; 440 mm total filtration length, 60 mm external diameter, and 40 mm internal diameter), filled with the Johnson Matthey catalyst pellets studied in this work. Those pellets were arranged inside the inert candle as an annular packed bed; the inner, empty cylindrical volume around the vertical axis of candle (20 mm diameter) allowed the conditioned syngas to leave the packed bed and flow toward the candle head along that axis (see ref (24) for further details and the graphical abstract of this work). Consequently, the external and internal radii of the catalytic packed-bed equaled 20 and 10 mm, respectively.

In this section, that catalytic annular packed bed was modeled while carrying out the reforming of hydrocarbons contained in the raw syngas, which rises from the fluidized bed beneath the filtering-catalytic candle.

As a first assumption, the syngas entering the annular packed-bed of catalyst pellets (i.e., at the external radius of 20 mm) is particulate-free, since the Pall Filter systems GmbH candles ensure more than 99.99% efficiency of solid particle removal.^24,41^

Table 4 summarizes the specifications of raw syngas from gasification tests by Savuto et al.^24^ The experiment with an empty Al_2_O_3_ candle (i.e., without the catalytic filling) in the gasifier freeboard provided typical flow rate and composition of the depulverized syngas at the entrance of the catalytic annular packed-bed (Table 4). It is worth noting that Savuto et al.^24^ did not report a detailed H_2_S quantification in the product syngas. However, in a previous work,^16^ dealing with steam gasification tests performed with the same rig and the same biomass type, H_2_S content in the dry product gas was found close to 45 ppm_v_, corresponding to 33 ppm_v_ in the syngas-containing steam of their tests.^16,22^ As a result, we assumed this value in our calculations (Table 4).

**Table 4:** Process Conditions and Results of Gasification Tests^a^

	empty candle	filtering-catalytic candle
Process Conditions		
*P* [atm]	1	1
average candle *T* [°C]	790	775^b^
*W* [g]	0	563.80
Gasification Inlet		
face filtration velocity [cm s^–1^]^c^	2.8	
N_2_ [mol h^–1^]	48.9	
Syngas Outlet		
steam [mol h^–1^]	15.2	
H_2_ [vol %_dryN_2_-free_]	40.6 ± 0.6	54.0 ± 0.6
CO [vol %_dryN_2_-free_]	29.2 ± 0.4	29.8 ± 0.2
CO_2_ [vol %_dryN_2_-free_]	21.2 ± 0.4	15.0 ± 0.6
CH_4_ [vol %_dryN_2_-free_]	9.0 ± 0.3	1.2 ± 0.2
tar outlet [mg Nm^–3^_dryN_2_-free_]	3276	357^d^
benzene outlet [mg Nm^–3^_dryN_2_-free_]	2439	74^d^
H_2_S [ppm_v_]	33	33

a Data adapted from Savuto et al.^24^

b Average temperature in the filter candle, calculated as suggested by Savuto et al.^24^

c Volumetric flow rate/external lateral cylindric surface.

d Tar and benzene concentrations obtained by averaging data in Table 3 of Savuto et al.^24^

With regard to the detailed tar composition in this inlet stream, the following species were detected in the syngas produced during the empty-candle test,^24^ ranging between 1 and 3 aromatic rings and reported in the order of decreasing abundance (see section S2 of the Supporting Information): toluene, naphthalene, acenaphthylene, styrene, pyrene, indene, biphenyl, anthracene, fluorene, phenanthrene, and fluoranthene. According to their quantification in section S2 of Supporting Information, the lumping into the pseudocomponent *C*_tar_ resulted in an *h*/*c* index of 0.9 in the formula CH_(*h*/*c*)_, which is close to that of the synthetic tar mixture used in the laboratory-scale tests (*h*/*c* = 1.0, Table 2).

In addition to tar compounds, CH_4_ and benzene were detected in the outlet stream of gasification tests in Table 4: In the syngas treated with the candle containing the annular catalytic packed bed, CH_4_ and benzene were less concentrated, while H_2_ concentration was higher; this suggested that syngas conditioning also involved steam methane reforming (SMR, reaction R3) and steam reforming of benzene (reaction R4):
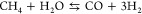
R3
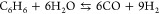
R4As a result, the catalytic packed bed was modeled as an isothermal, laterally fed annular PFR, involving reactions R1–R4.

The raw depulverized syngas is fed at the external PFR cylindrical lateral surface (corresponding to the interface between Al_2_O_3_ candle and the pellets); the syngas flows radially through the pellets. Meanwhile, the conversion of hydrocarbon occurs; the reformed syngas leaves the bed at its inner cylindrical lateral surface (see ref (24) for further details and the graphical abstract of this work). Equation 14 describes the resulting mole balance for the generic gaseous species *i*, flowing radially trough the annular packed bed, with its overall reaction rate defined by eq 15.
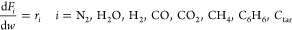
14
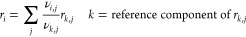
15In addition to the lumped kinetic law for the rate of reaction R1 referred to *C*_tar_ (*r*_*C*_tar_,1_), kinetic laws were also assumed from the literature for the remaining reactions. As previously done by this research team,^42,43^ Numaguchi and Kikuchi’s kinetic laws^44^ were assumed for both *r*_CO,2_ (rate of WGS referred to CO, reaction R2) and *r*_CH_4_,3_ (rate of SMR referred to CH_4_, reaction R3). The rate of benzene steam reforming (reaction R4), *r*_C_6_H_6_,4_, was described by the kinetic law proposed by Depner and Jess,^30^ which involved an adsorption term to take into account sulfur deactivation.^30^ The chance of using correction factors for literature kinetics was taken into account, in order to consider the use of a catalyst different from those utilized in the original papers.

#### 3.3.2 Syngas Cleaning: Comparison between Simulations and Experimental Data

The model developed in section 3.3.1 was applied to the case of the catalytic annular packed bed inside the filtering-catalytic candle (Table 4). The molar flow rates of the empty-candle test (Table 4) were assumed as the feed of the annular packed bed. The consequent WHSV and WHSV_*C*_tar__ equaled 174.3 mol h^–1^ kg_cat_^–1^ and 0.34 mol_*C*_tar__ h^–1^ kg_cat_^–1^, respectively; therefore, typical reaction conditions experienced by the Ni-catalyst pellets in the filtering-catalytic candles were less severe than those imposed in the packed-bed tests at laboratory scale (see Table 2 and section 3.1).

The mole balances (eq 14, with eq 15) were implemented in MATLAB and numerically integrated by the “ode45” routine. Table 5 summarizes the numerical results of this simulation: a comparison of calculated outlet in Table 5 with experimental data in Table 4 revealed a fair agreement with the gasification experiment of Savuto et al.^24^

**Table 5:** Syngas Composition at Inlet and Outlet of the Catalytic Layer Inside the Filter Candle Simulated as a Laterally-Fed Annular PFR^a^

	inlet	outlet
N_2_ [mol h^–1^]	48.9	48.9
steam [mol h^–1^]	15.2	11.8
H_2_ [vol %_dryN_2_-free_]	40.6	52.6
CO [vol %_dryN_2_-free_]	29.2	28.8
CO_2_ [vol %_dryN_2_-free_]	21.2	17.7
CH_4_ [vol %_dryN_2_-free_]	9.0	0.9
CH_4_ conversion [%]		86.9
tar [mg Nm^–3^_dryN_2_-free_]	3276	362
tar conversion [%]		85.9
benzene [mg Nm^–3^_dryN_2_-free_]	2439	80
benzene conversion [%]		95.8

a Conditions: *T* = 775 °C, *P* = 1 atm, *W* = 563.80 g, 33 ppm_v_ H_2_S.

As to the rate of *C*_tar_ steam reforming (*r*_*C*_tar_,1_), the lumped approach used to describe tar chemistry and kinetics turned out to be successful: In the clean syngas, 362 mg Nm^–3^_dryN_2_-free_ was predicted (Table 5), very close to the experimental 357 mg Nm^–3^_dryN_2_-free_ (Table 4). Noticeably, in order to obtain this result, neither the lumped kinetic law for *C*_tar_ steam reforming (eqs 5–8) nor its kinetic parameters in Table 3 had to be tuned.

It is worth stressing here that WHSV and WHSV_*C*_tar__ of the simulated cleaning process were somewhat different from those of the experiments in the packed-bed rig (Table 2); this further points to the reliability of the approach proposed in this work to estimate tar reforming in a hot gas catalytic treatment and adds to the obvious advantages linked to the use of a small amount of catalyst and a lab-scale experimental setup.

The rate laws taken from the literature for reactions R3 and R4 had to be tuned. In order to match the experimental composition of CH_4_ and benzene in the clean syngas leaving the filtering-catalytic candle (Table 4), multiplying factors equal to 2.3 × 10^–2^ and 4.5 were used for *r*_CH_4_,3_ and *r*_C_6_H_6_,4_, respectively. With regard to the reduction of reaction R3’s rate (*r*_CH_4_,3_), the sulfur deactivation is certainly a contributing factor: The Numaguchi and Kickuchi’s law adopted here does not involve any sulfur deactivation term,^44^ while sulfur deactivation of Ni sites in the case of SMR (reaction R3) was experimentally evaluated as the most pronounced, among an investigated group counting naphthalene, benzene, CH_4_, and NH_3_.^30^ In addition, for methane steam reforming catalyzed by pellets, it is well-known that an effectiveness factor of the order 10^–2^ is reasonable.^45^ As to reaction R4’s rate (*r*_C_6_H_6_,3_), the tuning could be related to the quite important differences in the Ni load and support nature between the catalyst studied in this work and that investigated by Depner and Jess.^30^ As far as the WGS is concerned, no tuning of *r*_CO,2_ was carried out, since the outlet composition of cleaned syngas resulted close to its thermodynamic equilibrium.

These tuning operations regarding methane and benzene do not affect the good prediction of tar removal by the lumped expression used for the reaction rate of tar steam reforming (*r*_*C*_tar_,1_). The contribution of *C*_tar_ decomposition to the variation of H_2_, CO, and CO_2_ flow rates and concentrations is negligible when compared to that due to SMR (reaction R3), since the flow rate of CH_4_ at packed bed inlet (3.07 mol h^–1^) is much higher than that of *C*_tar_ (0.19 mol h^–1^), by 1 order of magnitude. In contrast, the inlet flow rate of benzene was 0.02 mol h^–1^, equivalent to 0.12 mol h^–1^ of carbon atoms, resulting in the same order of the above-mentioned *C*_tar_ inlet. Provided that CH_4_ is fed to the Ni catalyst in much higher quantities than benzene and tars, predictions regarding H_2_, CO, CO_2_, and CH_4_ are not appropriate indicators to assess the reliability of the lumped kinetic approach; only tar quantification is.

**Figure 7:**
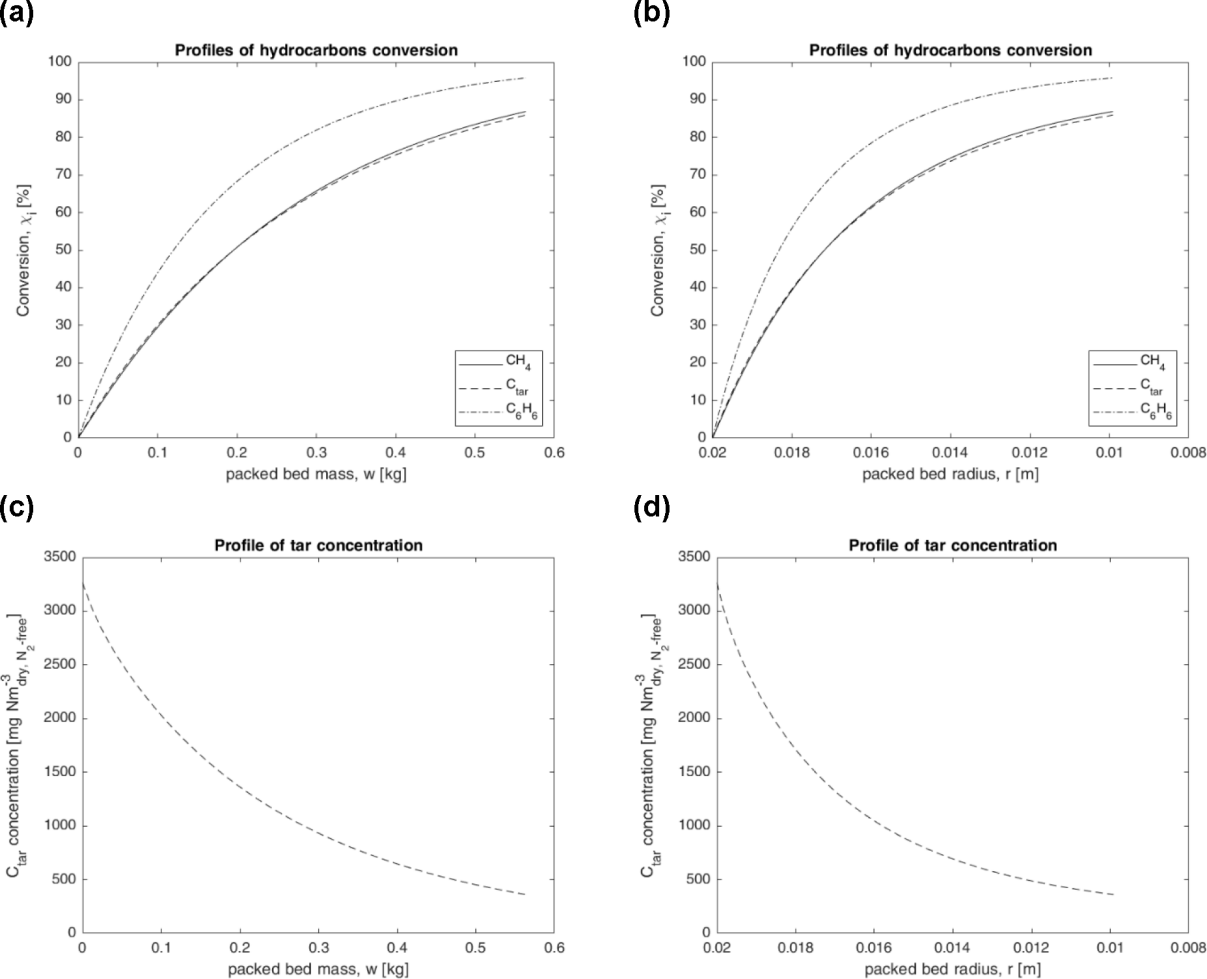
Simulation results predicted by the model of the laterally fed cylindrical PFR (*T* = 775 °C, *P* = 1 atm, *W* = 563.80 g, 33 ppm_v_ H_2_S): profiles of hydrocarbons conversion (a, b) and tar concentration (c, d).

**Figure 8:**
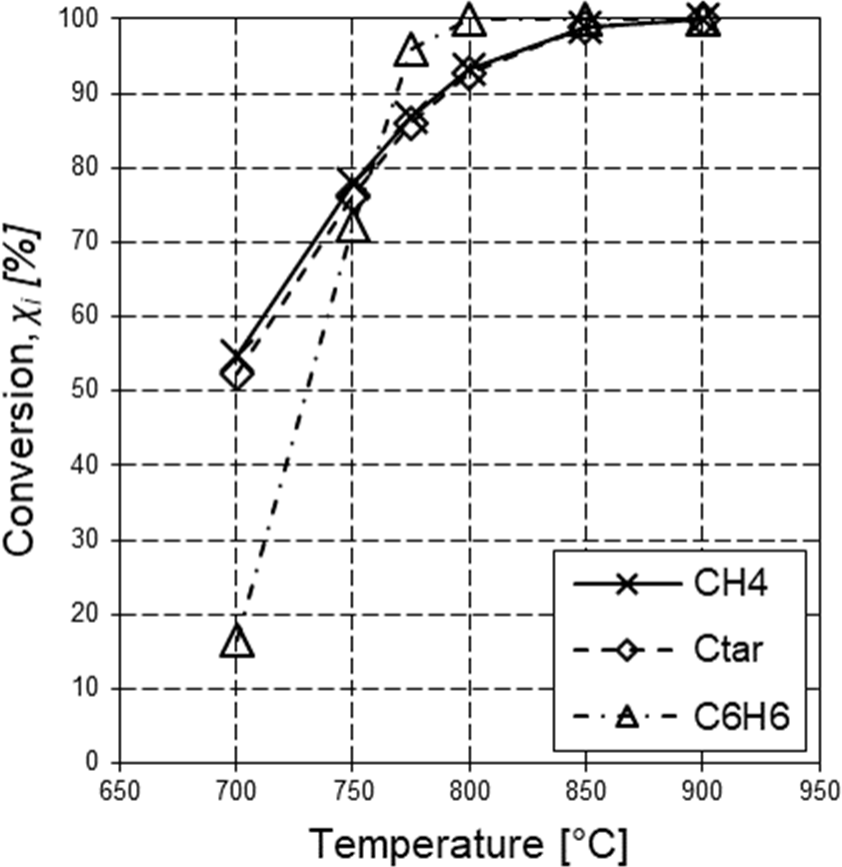
Simulation results predicted by the model of the laterally fed cylindrical PFR (*P* = 1 atm, *W* = 563.80 g, 33 ppm_v_ H_2_S): hydrocarbon conversions as functions of temperature.

Thanks to the just discussed kinetic laws, the model of the laterally fed cylindrical PFR produced several trends concerning the performance of the annular packed bed, as functions of its mass or its radius (Figure 7). In addition, that model was used to predict the performance of the catalytic annular packed-bed at different temperatures in the range 700–900 °C, by simulating the same process (Table 4) and inlet (Table 5) conditions of the case just discussed above (for previsions at *T* > 800 °C the lumped kinetic law for *C*_tar_ was extrapolated by data in the range 700–800 °C). Results (Figure 8) are in fair agreement with the experimental findings by Ma et al.,^29^ who carried out steam reforming tests of tar key compounds on Ni catalyst, in the presence of H_2_S and at typical process conditions experienced by filtering-catalytic candles during in situ syngas cleaning: they found a significant increase of hydrocarbons conversion as the temperature was increased, reaching almost complete removal of tarry molecules at 900 °C.^29^

## 4 Conclusions

This work stems from the need to improve the understanding of tar removal that takes place in filtering-catalytic candles, used for the in situ hot syngas cleaning in the freeboard of a pilot-scale fluidized-bed steam gasifier. The catalytic phase in those candles was confined in an annular packed-bed made of commercial Ni-catalyst pellets, supplied by Johnson Matthey. These pellets were proved to act satisfactorily by tests in the above-mentioned gasifier (Savuto et al.),^24^ in terms of tar removal from the real syngas produced by biomass steam gasification. In contrast, the simultaneity of gasification and syngas cleaning, the pilot scale, and the allowed measurements did not enable a thorough, dedicated observation of phenomena occurring just in the filtering-catalytic candle. The present study aimed to improve the insight into these phenomena.

Dedicated reactivity tests were carried out at laboratory scale in a fully controlled packed-bed reactor: Steam and a synthetic tar mixture (naphthalene, toluene, thiophene in traces) were fed as reactants and converted by steam reforming on an active packed bed made of the same commercial Ni-catalyst pellets, previously used in the filtering-catalytic candles.

This experimental campaign allowed inference of a lumped kinetic law (pseudo-first-order) for the steam reforming of tar, which also included the deactivation of Ni by sulfur species. The so obtained kinetic parameters were in line with the literature about steam reforming of tar key compounds on Ni catalyst.

The lumping process consisted of (i) reducing the tar mixture into a representative monocarbonic pseudocomponent with formula CH_(*h*/*c*)_ and (ii) considering the tar steam reforming as governed by the reaction between this CH_(*h*/*c*)_ group and steam, which eventually forms hydrogen and carbon oxides, also thanks to the simultaneous occurrence of WGS. The procedure to reduce a tar mixture into the formula CH_(*h*/*c*)_ was totally general, as well as the formulation of the kinetic law for the steam reforming of this pseudocomponent. This enabled the extension of the lumped kinetics, obtained for a synthetic tar mixture, to the case of real tar reforming during syngas cleaning in the filtering-catalytic candle.

This extension was performed and validated by implementing the lumped kinetic law in a mathematical model of the annular catalytic packed bed inside a filter candle, radially fed with the raw syngas stream rising from the fluidized bed of the biomass steam gasifier. The actual syngas also contained methane and benzene, so their steam reforming reactions were included in the model by means of the respective kinetic laws taken from the literature; WGS was also included. A simulation was carried out of an actual case of syngas cleaning in the pilot gasifier equipped with a filtering-catalytic candle. The numerical results were in fair agreement with experimental findings, especially with regard to tar removal, and noticeably without any further tuning of the lumped kinetic law for CH_(*h*/*c*)_ steam reforming.

It is worth stressing here that the lumping procedure allowed a fair prediction of the behavior of a complex tar mixture, made up of 11 different hydrocarbons and produced during a real thermochemical process, by means of laboratory-scale experiments with a much simpler synthetic tar mixture, made up of only two main key compounds (i.e., toluene and naphthalene).

As a result, an effective and general procedure was proposed, carried out, and validated. This procedure provided simple and reliable tools for the straightforward investigation of tar steam reforming during hot syngas cleaning and conditioning, strictly integrated with a gasification process.

## Supporting Information Available

Stoichiometric table; *C*_tar_ molar balance for a steady-state PFR; quantification of tarry molecules in the empty-candle test (PDF)

## Abbreviations

BLAZE: Biomass Low-cost Advanced Zero Emission
EU: European Union
EUBCE: EUropean Biomass Conference and Exhibition
GC: Gas-Chromatograph
Ni: nickel
LHS: Left-Hand Side
PAH: PolyAromatic Hydrocarbons
PFR: Plug Flow Reactor
SMR: Steam Methane Reforming
USA: United State of America
WGS: Water Gas Shift
WHSV: Weight Hourly Space Velocity
WHSV_*C*_tar__: Weight Hourly Space Velocity referred to *C*_tar_

## Symbols

*c*: index in molecular formula of *C*_tar_, eq 3
*C*_*i*_: molar concentration of gaseous species *i*, mol m^–3^
*E*_a,*j*_: activation energy of Reaction *j*, kJ mol^–1^, eq 7
*F*_*i*_: molar flow rate of species *i*, mol h^–1^
*h*: index in molecular formula of *C*_tar_, eq 4
*k*_*i*,*j*_: specific reaction rate of Reaction *j*, referred to species *i*, m^3^ kg_cat_^–1^ h^–1^
*K*_S_: H_2_S adsorption equilibrium constant, atm^–1^, eq 8
*m*_*i*_: number of H atoms in the formula of hydrocarbon *i*, eq 3
*N*: number of hydrocarbons in tars mixture, eq 3 and eq 4
*n*_*i*_: number of C atoms in the formula of hydrocarbon *i*, eq 4
*p*_*S*_: partial pressure of sulfur species, atm, eq 6
*P*: pressure, atm
*R*: universal gas constant, 8.314 × 10^–3^ kJ mol^–1^ K^–1^
*R*^2^: coefficient of determination
*r*_*i*_: overall reaction rate of species *i*, mol kg_cat_^–1^ min^–1^, eq 14 and eq 15
*r*_*i*,*j*_: reaction rate of Reaction *j* referred to species *i*, mol kg_cat_^–1^ min^–1^, eq 15
*T*: temperature, K
*t*: time, h
*w*: packed bed mass (variable), kg
*W*: total packed bed mass, kg
*x*_tar,*i*_: molar fraction of hydrocarbon *i* in tar mixture, mol mol^–1^, eq 3 and eq 4
*Y*_*i*_: molar percentage dry, dilution-free of gaseous species *i*, vol % _dry,dil.-free_
*y*_*i*_: molar percentages of species *i*, measured by ABB system, vol %
**Greek Symbols**
α: molar steam to carbon ratio, mol mol^–1^
β: molar N_2_ to steam ration, mol mol^–1^
Δ*H*_S_: enthalpy of adsorption of H_2_S on Ni sites, referred to *C*_tar_, kJ mol^–1^
*ν*_*i*,*j*_: stoichiometric coefficient of species *i* in reaction *j* (<0 for reactants, >0 for products)
τ: proper time interval of steady-state steam reforming, h
χ_*i*_: conversion of species *i*
**Subscripts and Superscripts**
app: apparent
in: inlet
out: outlet
S: sulfur species
0: pre-exponential factor
