# Supplementary material for: Kinetic Characterization of Tar Reforming on Commercial Ni-Catalyst Pellets Used for In Situ Syngas Cleaning in Biomass Gasification: Experiments and Simulations under Process Conditions
Source: Ind Eng Chem Res. 2020 Dec 16;60(18):6421–34. doi: 10.1021/acs.iecr.0c05131 (PMC8154441; doi:10.1021/acs.iecr.0c05131)
Supplement: Supplementary file 1 — ie0c05131_si_001.pdf [file ie0c05131_si_001.pdf]

Supporting Information

Kinetic Characterization of Tar Reforming on Commercial Ni Catalyst Pellets Used for In Situ Syngas Cleaning in Biomass Gasification: Experiments and Simulations at Process Conditions

Andrea Di Giuliano[1], Pier Ugo Foscolo[1], Andrea Di Carlo[1], Andrew Steele[2], Katia Gallucci[1].*

[1] University of L'Aquila, Department of Industrial and Computer Engineering and Economics, Piazzale E. Pontieri 1 - loc. Monteluco di Roio, 67100 L'Aquila, Italy
[2] Johnson Matthey Technology Centre (JMTC), Blount's Court, Sonning Common, Reading RG4 9NH, United Kingdom

Enrico Tronconi Festschrift

Corresponding Author:
*andrea.digiuliano@univaq.it
University of L'Aquila, Department of Industrial and Computer Engineering and Economics, Piazzale E. Pontieri 1 - loc. Monteluco di Roio, 67100 L'Aquila, Italy
tel. +39 0862 434213

S1. Stoichiometric Table and C_{tar} Molar Balance for a Steady-State PFR

This section describes how Equation 9 and Equation 10 of the main manuscript were obtained.

Let us consider the catalytic pellets which constitute the active packed-bed in the test rig described in subsection 2.2.

Equation S1 is the mole balance for the C_{tar} pseudo-component, flowing through the packed-bed modelled as a PFR in steady-state.

$$\frac{dF_{C_{tar}}}{dw} = -\boldsymbol{r_{C_{tar,1}}} \qquad \text{Equation S1}$$

The right-hand side of Equation S1 is defined by Equation 5 in the main manuscript, as a function of C_{tar} molar concentration (C_{Ctar}). C_{tar} flow rate (F_{Ctar}) and C_{Ctar} are variable along the packed-bed (i.e. they depend on the packed bed mass w), so we need to express them in terms of experimentally measurable quantities. This was done by means of manipulations summarized in **Table S1** and the ideal gas law [1], obtaining Equation S2. According to **Table S1** and Equation S2, the balance in Equation S1 can be rewritten as shown in Equation 9 of the main manuscript, and then integrated by separation of variables to obtain Equation 10.

Table S1. Stoichiometric table for Reaction 1, as a function of C_{tar} conversion (χ_{Ctar}), h, c, inlet C_{tar} flow rate ($F_{Ctar,in}$), inlet molar steam to carbon ratio (α_{in}), and inlet molar N_2 to steam ratio (β_{in}).

STOICHIOMETRIC TABLE					
	**$CH_{h/c}$**	**H_2O**	**N_2**	**CO**	**H_2**
**initial**	$F_{C_{tar},in}$	$F_{C_{tar},in}\alpha_{in}$	$F_{C_{tar},in}\alpha_{in}\beta_{in}$	0	0
**change**	$-F_{C_{tar},in}\,\chi_{C_{tar}}$	$-F_{C_{tar},in}\,\chi_{C_{tar}}$	0	$+F_{C_{tar},in}\,\chi_{C_{tar}}$	$+\left(1+\frac{h}{2c}\right)F_{C_{tar},in}\,\chi_{C_{tar}}$
**remaining**	$F_{C_{tar},in}\left(1-\chi_{C_{tar}}\right)=F_{C_{tar}}$	$F_{C_{tar},in}\left(\alpha_{in}-\chi_{C_{tar}}\right)$	$F_{C_{tar},in}\alpha_{in}\beta_{in}$	$F_{C_{tar},in}\,\chi_{C_{tar}}$	$\left(1+\frac{h}{2c}\right)F_{C_{tar},in}\,\chi_{C_{tar}}$
**sum of remaining**		$F_{tot}=F_{C_{tar},in}\left(1+\alpha_{in}\left(1+\beta_{in}\right)+\left(\frac{h}{2c}\right)\chi_{C_{tar}}\right)$			

$$C_{C_{tar}} = \left(\frac{P}{R\,T}\right)\frac{F_{C_{tar}}}{F_{tot}} = \left(\frac{P}{R\,T}\right)\frac{F_{C_{tar},in}\left(1-\chi_{C_{tar}}\right)}{F_{C_{tar},in}\left(1\;+\;\alpha_{in}\left(1\;+\;\beta_{in}\right)+\left(\frac{h}{2c}\right)\chi_{C_{tar}}\right)} \qquad \text{Equation S2}$$

S2. Quantification of Tarry Molecules in the Empty-Candle Test

As stated by Savuto et al. [2], tar was analyzed and quantified by gas chromatography-mass spectrometry (GC-MS), but detailed numerical results were not reported in that work. **Table S2** summarizes GC-MS results [3] concerning the tar molecules in the syngas produced by the steam gasification test with the empty-candle, detailed in Table 4 of the main manuscript. Quantities in **Table S2** were lumped to obtain the C_{tar} pseudo-component which represents the tar mixture fed to the annular packed-bed simulated in subsection 3.3 of the main manuscript.

Table S2. Tar compounds generated during the empty-candle steam gasification test (data from [3]: concentrations in mg $Nm^{-3}{}_{dry,N2\text{-}free}$

Tar compound	Formula	Concentration	
		[mg $Nm^{-3}{}_{dry,N2\text{-}free}$]	[mmol $Nm^{-3}{}_{dry,N2\text{-}free}$]
toluene	C_7H_8	1272.30	13.81
naphthalene	$C_{10}H_8$	615.63	4.80
acenapthylene	$C_{12}H_8$	382.37	2.51
styrene	C_8H_8	224.25	2.15
pyrene	$C_{16}H_{10}$	376.25	1.86
indene	C_9H_8	211.50	1.82
biphenyl	$C_{12}H_{10}$	47.94	0.31
anthracene	$C_{14}H_{10}$	54.07	0.30
fluorene	$C_{12}H_{10}$	48.19	0.29
phenathrene	$C_{14}H_{10}$	19.72	0.11
fluoranthene	$C_{16}H_{10}$	19.89	0.10
phenol	C_6H_6O	not detected	not detected
xylene	C_8H_{10}	not detected	not detected

REFERENCES

(1) Fogler, H. S. *Elements of Chemical Reaction Engineering*, 4th ed.; Prentice Hall: Westford, Massachusets, 2005.

(2) Savuto, E.; Di Carlo, A.; Steele, A.; Heidenreich, S.; Gallucci, K.; Rapagnà, S. Syngas Conditioning by Ceramic Filter Candles Filled with Catalyst Pellets and Placed inside the Freeboard of a Fluidized Bed Steam Gasifier. *Fuel Process. Technol.* **2019**, *191*, 44–53. https://doi.org/10.1016/j.fuproc.2019.03.018.

(3) Savuto, E. Università Degli Studi Dell’Aquila, Internal report. 2018.
